# Analyzing the scale dependent effect of urban building morphology on land surface temperature using random forest algorithm

**DOI:** 10.1038/s41598-023-46437-w

**Published:** 2023-11-07

**Authors:** Weiqun Han

**Affiliations:** School of Management, Wuhan Donghu University, N301, Wenhua Avenue, Jiangxia District, Wuhan, 430212 People’s Republic of China

**Keywords:** Environmental impact, Climate-change mitigation

## Abstract

With continuous urban densification, revealing impacts of urban structures on thermal environment is necessary for climate adaptive design. In this study, random forest and partial difference plots were employed to depict the relative importance and interdependent effects of complex building morphology to land surface temperature (LST) variability. The six spatial factors of building density (BD), mean building height (MBH), building height difference (BHD), floor area ratio (FAR), building volume density (BVD) and mean compactness factor (MCF) were calculated at grids of 90, 300, 600 and 900 m. The results showed that BD, MCF and MBH exerted stable and significant impacts on LST with the highest prediction accuracy at 600 m neighborhood scale, and FAR and BVD were the least correlated to LST changes. Meanwhile, the influencing factors presented different correlation patterns with LST. Among them, the increase of BD had a positive linear effect on LST. MCF and MBH were nonlinearly correlated with the LST variation, and their threshold values of cooling effect were also identified. In addition to controlling BD, it also suggested that comprehensively arranging more small-volume buildings as well as increasing building height to enlarge shadow coverage were more conducive to ground heat mitigation.

## Introduction

Although urban areas occupy a small portion of the land territory, it is home to more than half of the world's population^1^. Under the conditions of rapid increase of urban population and limited land resources for construction, cities are sprawling rapidly both in the horizontal and vertical dimension, presenting the highly complex urban textures. Accordingly, large areas of original natural environment of woodland, wetlands and water bodies have been replaced by impermeable built-up surface, which intensively alters the surface energy budget and leads to the formation and intensification of urban heat island effect^2^. Therefore, within the compact modern cities, revealing how the urban planning and design processes affect the spatial heterogeneity of thermal environment and proposing relevant climate adaptive measures has become a major issue for sustainable living environment development^3^.

Satellite-based land surface temperature (LST) has been extensively applied to analyze the urban thermal environments due to its high spatial resolution and ease of use. Previous studies mainly disclosed the thermal effects of the urban landscape composition and landscape patterns. The landscape composition is usually expressed as a ratio of different subsurface types, and since each landscape component has corresponding albedo and thermal characteristics, their effects on surface temperature differ^4^. It has been widely proved that the area proportion of vegetation and water bodies is negatively correlated with surface temperature, while the area proportion of impermeable surface such as building groups and roads is positively correlated with surface temperature^5–7^. For landscape patterns, the variation of shapes and spatial distribution of landscape patches can further affect the efficiency of energy exchange processes, thus changing the surface heat flux^8^. Correspondingly, landscape metrics such as edge density (ED), patch density (PD), and Shannon’s diversity index (SHDI) were used to quantify the spatial layout of landscape elements such as urban green spaces and buildings, thus exploring their impacts on surface temperature^9–11^. In addition, some studies investigated the thermal environments among the underlying surface with various physical properties or color^12^, and assisted the cool materials selection. With the increasing complexity of urban land use pattern, the urban spatial morphology is also characterized by various building layout and undulating high-rise buildings^13^, making the applicability of the above-mentioned conclusions which focused on the thermal effects of two-dimensional landscape characteristics limited^14^. It is no doubt that an in-depth understanding of the interactions between the complex urban structures and thermal environments can better instruct the urban planning for climate change mitigation^15^.

Another perspective comprehensively investigated the influences of 2D and 3D spatial morphological features on urban thermal environment among the building clusters, but the results were sometimes inconsistent or even contradictory. For instance, Zheng et al. found a significant negative correlation between building height and surface temperature, while the building area density was the opposite^16^. Another research indicated that spatial parameters such as average building height and standard deviation of building height, which described the vertical configuration of buildings, played a dominant role in predicting surface temperature^17^. In the contrast, Srivanit and Kazunori analyzed the influence of 13 spatial parameters on surface temperature and emphasized that three-dimensional spatial parameters have a greater thermal effect, such as building volume ratio. Several composite parameters such as sky view factor (SVF), roughness length (Z_0_) and façade area index (FAI) were also used to incorporate the thermal considerations into the complex building morphology analysis^18^. The related studies shown that these parameters, which quantitatively described the geometry of three-dimensional space, still had a controversial impact on the thermal environment. In specific, some studies suggested that he high SVF mean more incoming solar radiation into the surface resulting to higher temperature during the daytime^19,20^. The opposite point was that the high SVF was conducive to improving the air ventilation and mitigating the surface temperature, accordingly^21,22^.

The existing studies provided scientific references for urban morphology control and management based on thermal environment optimization, but there are some limitations that need to be further addressed. First, the simple linear models are still commonly used to evaluate the influence of spatial factors on LST. However, this parametric regression model can be meaningful only when the linear relationship between the target variable and the LST is pre-known during the modeling process^4^. In fact, the actual relationships between urban morphology and LST is still unclear. More importantly, the urban spatial forms are interdependent, making it more intricate to analyze the combined effects and priorities of multiple factors on thermal environment^23^. Second, although there have been proposed various spatial parameters to describe the urban morphology, the thermal impact caused by spatial heterogeneity of building vertical configuration and building layout has not been fully paid attention to. Furthermore, due to the scarcity of urban land resources, identifying an appropriate size of analysis unit is essential to assist the urban morphology control for thermal environment-based planning and design^24,25^. However, there lacks comprehensive assessments of the main factors and dynamic changes affecting LST within difference spatial scales, which is not conducive to maximizing the optimization of urban morphology to thermal environment improvement.

Taking Wuhan which is a highly urbanized city in China as an example, we used satellite remote sensing data to investigate the impacts of urban building forms on urban heat according to RF model. The main objectives are to: (1) quantitatively analyze the relative influences of heterogeneous building spatial features on LST among different scales; (2) recognize the marginal effects of the dominant factors on LST variability; and (3) explore the optimal size of spatial unit for investigating LST distribution as well as formulating the thermal environment-based building management strategies.

## Materials and methods

### Study area

**Figure 1:**
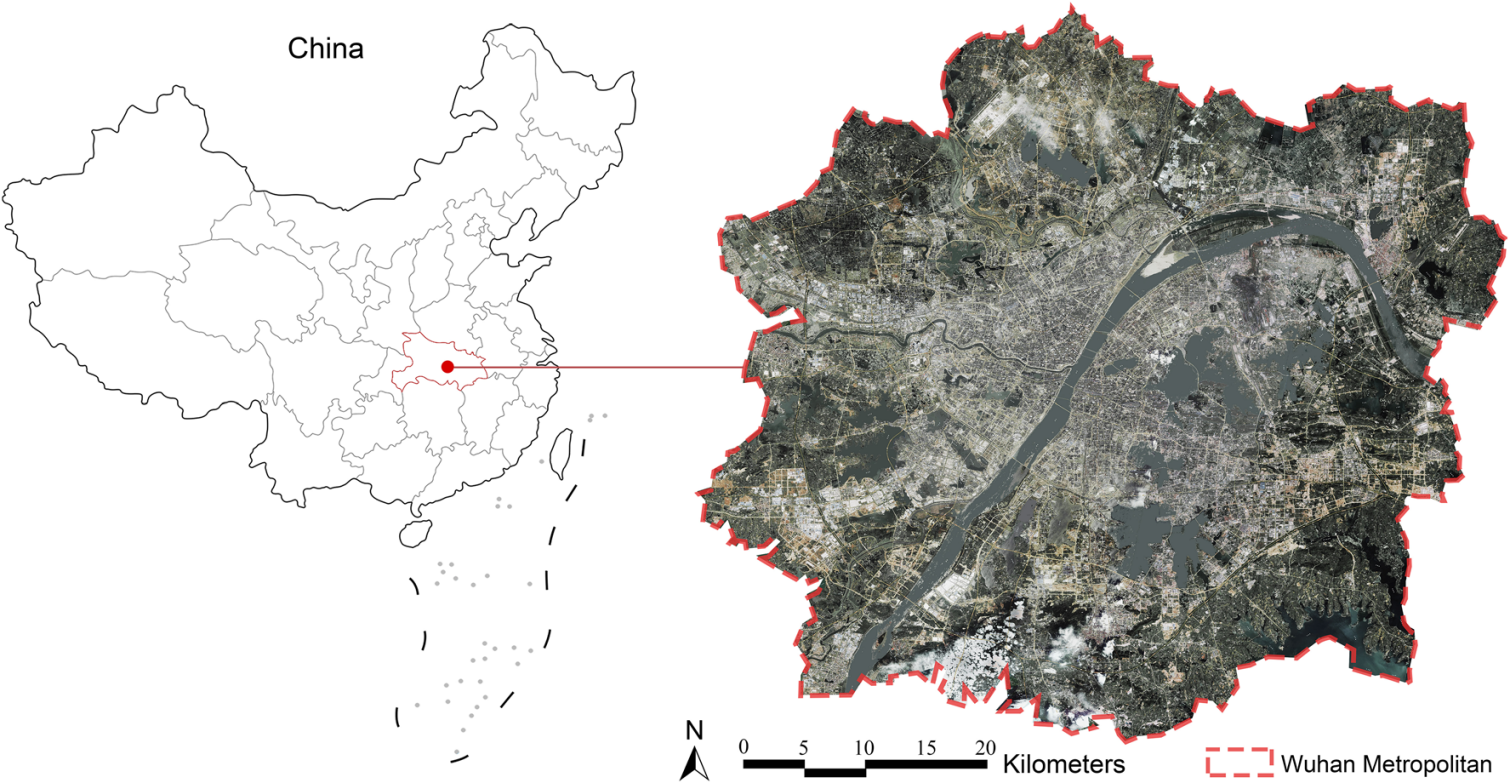
The location of WMA in Wuhan, China (Image was generated using ArcGIS 10.5 (https://www.esri.com/en-us/home) based on Google Remote Sensing images (http://www.gditu.net/)).

Wuhan is a megacity located in central China (30°35′ N, 114°17′ E), also known as one of China’s “furnace cities”. Wuhan has a subtropical monsoon climate (Koppen classification: Cf), characterized by long and hot summer. During the high-intensity urban densification and expansion process, Wuhan has formed a typical spatial structure of circular and layered development. Among them, the Wuhan metropolitan area (WMA) is our research area (Fig. 1), which is the main urban functional area and includes the central city and six new urban towns surrounding it. With eight million residents live in the area of 3261 km^2^, the compact built environment and numerous newly built high-rise buildings make the surface structures of WMA heterogeneous, resulting in a highly complex spatial distribution of the thermal environment. Thus, identifying the key building spatial forms on affecting surface thermal environment is a practically important part for guiding the development of mitigation strategies to better cope with the heat stress.

### LST data retrieval

The LST data were collected from Landsat 8 thermal infrared sensor (TIRS) images from the United States Geological Survey (USGS) at a spatial resolution of 30 m (http://www.usgs.gov/). During the summer of 2022 (June to September), WMA suffered the long-lasting hot heat wave weather, which posed the serious additional health threat to urban residents. Among the summer satellite samplings, the thermal imagery obtained in August 9th can better ensure the representative high temperature as well as the cloudless weather background within the study area. Specifically, the atmosphere temperature was between 302.15 K and 312.15 K, and it was about 309.15 K at the time of image acquisition with relative humidity of 72% and land cloud cover of 1.42%. The process of LST data retrieval was carried out based on following steps. The surface temperature of the study area was inverted by thermal infrared band 10 (TIRS10) using the mono-window algorithm after radiometric calibration and atmospheric correction. The TIRS10 band can be converted to brightness temperature using the following equation:

1 $${\mathrm{T}}=\frac{{\mathrm{K}}_{2}}{{\mathrm{ln}}\left(\frac{{\mathrm{K}}_{1}}{{\mathrm{M}}_{10}{\mathrm{Q}}_{\mathrm{cal}}+{\mathrm{A}}_{\mathrm{L}}+1}\right)^{{\prime}}}$$

where T is the brightness temperature of TIRS10, ML is the tuning factor of TIRS10, Q_cal_ is the number of digits of Landsat 8 image, A_L_ is the tuning parameter of TIRS10, K_1_ = 774.89 W/m^2^-sr-µm, K_2_ = 1321.08 K. Finally, from Eq. (2), the LST can be obtained:

2 $${\mathrm{LST}}=\upgamma \left[{\upvarepsilon }^{-1}\left(\frac{1}{\uptau }{\mathrm{L}}+\left(-{\mathrm{L}}\downarrow -\frac{{\mathrm{L}}\uparrow }{\uptau }\right)\right)+{\mathrm{L}}\downarrow \right]+\updelta$$

3 $$\upgamma =\frac{{\mathrm{T}}^{2}}{{\mathrm{b}}_{10}\mathrm{L}^{\prime}}$$

4 $$\updelta ={\mathrm{T}}-\frac{{\mathrm{T}}^{2}}{{\mathrm{b}}_{10}^{{\prime}}}$$

where ε is the surface emissivity calculated from the normalized vegetation index (NDVI)^26^, τ is the atmospheric transmittance, and L↑ and L↓ are the atmospheric upward and downward radiation intensities, respectively, with data from the official Meteomanz website (http://www.meteomanz.com/, accessed August 9, 2022) and the Atmospheric Correction Parameter Calculator (https://atmcorr.gsfc.nasa.gov/, accessed 9/8/2022).

### Building spatial factors selection

**Figure 2:**
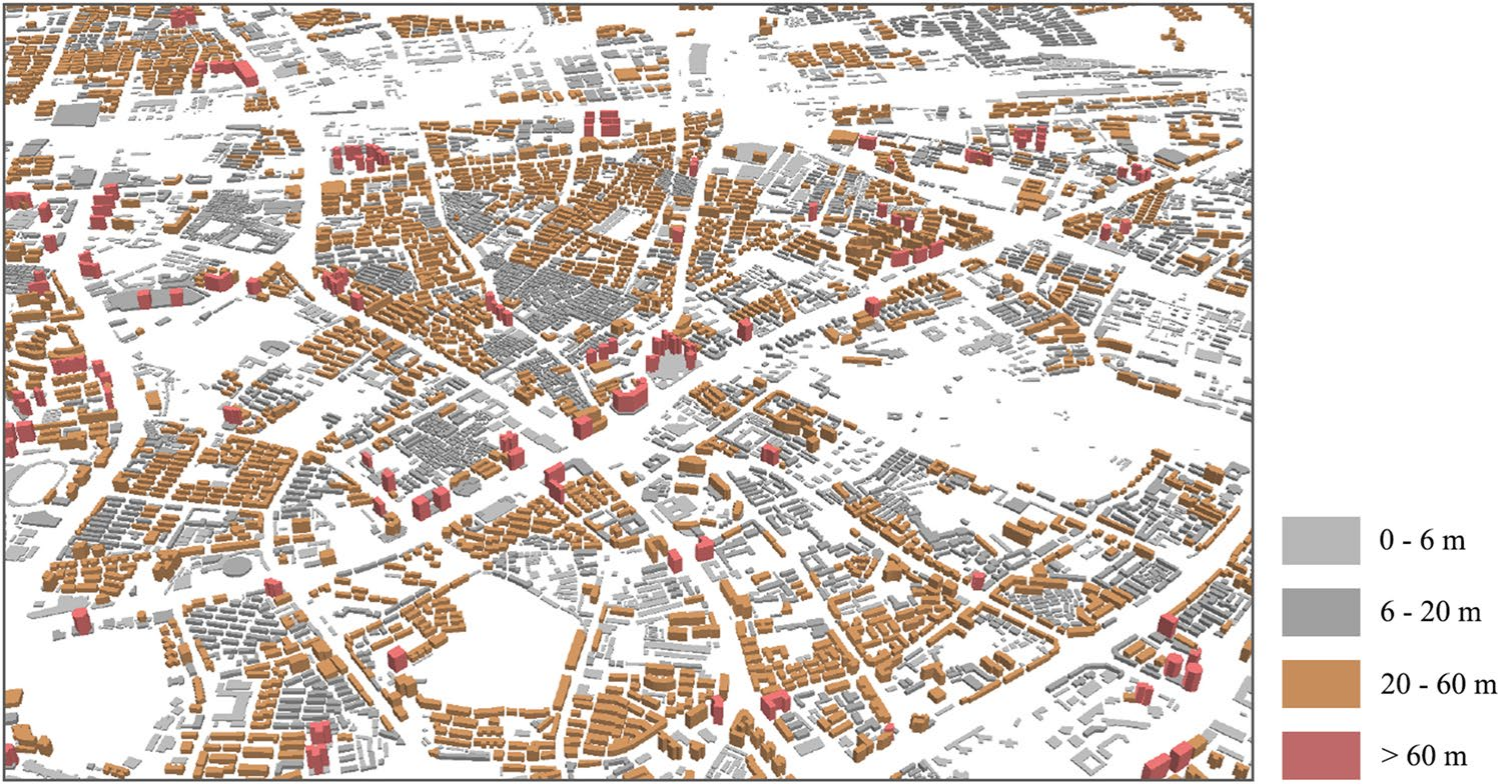
Building data of Wuhan rendered by building height.

The realistic urban building forms are always highly complex and diverse, containing the variations in spatial volume and geometric configuration. To quantitatively describe the spatial heterogeneity of building clusters, we chose building density (BD), mean building height (MBH), building height difference (BHD), floor area ratio (FAR), building volume density (BVD) and mean compactness factor (MCF), as presented in Table 1. The selection criteria of these parameters included: (1) they can effectively reflect the complex spatial characteristics of building forms at horizontal and vertical direction; (2) they can be intuitively understood and also met the practical needs of urban design; (3) they have been utilized in previous research. It should be noted that, the building height data was obtained from Baidu, Inc. (https://map.baidu.com/) with a resolution of 3 m and the data and preprocessing were completed under the WGS1984 coordinate system and UTM projection. Then, the fishnet tool (ArcGIS 10.5) was used to uniformly divide the study area and extract the high-precision building spatial data within each grid. In specific, we resampled 3 m building height data into 30 m by the nearest neighbor method (Fig. 2). Accordingly, we used grids of 90, 300, 600 and 900 m as the scale of analysis based on multiples of the resolution of the LST data generated by Landsat 8. These units corresponded to residential areas (90 m), urban blocks (300 m), neighborhood (600 m), and communities (900 m), so that comprehensively analyzed how the impact of building forms on the thermal environment can be affected along with the spatial scales.

**Table 1:** Summary of the selected building spatial form factors.

Variables	Abbr	Formula	Description
Building density	BD	$\frac{{\sum\nolimits_{i = 1}^{n} {A_{Bi} } }}{A}$	Where A_*Bi*_ is the base area of building, n is the number of buildings in a grid, and A is the area of the grid
Mean building height	MBH	$\frac{{\sum\nolimits_{i = 1}^{n} {V_{i} } }}{{\sum\nolimits_{i = 1}^{n} {A_{Bi} } }}$	Where V_*i*_ is the building volume*,* A_*Bi*_ is the base area of building
Building height difference	BHD	H_*max*_ − MBH	Where H_*max*_ is the height of the tallest building and MBH is the mean building height
Floor area ratio	FAR	$\frac{{\sum\nolimits_{i = 1}^{n} {A_{Ti} } }}{A}$	Where A_Ti_ is the total area of building and A is the grid area
Building volume density	BVD	$\frac{{\sum\nolimits_{i = 1}^{n} {V_{i} } }}{A}$	Where V_*i*_ is the volume of building and A is the grid area
Mean compactness factor	MCF	$\frac{{\sum\nolimits_{i = 1}^{n} {A_{Ei} } }}{{\sum\nolimits_{i = 1}^{n} {V_{i} } }}$	Where A_*Ei*_ is the area of building envelope, V_*i*_ is the building volume

### Random forest model

Random Forest (RF) is a non-parametric machine learning (ML) method that has been applied for prediction and regression analysis. The basis of the RF model is the decision trees. Each decision tree is established through randomly selected training samples and randomly selected predictor variables, which are combined to generate the final predicted values^27^. In a comparison of other ML algorithms, the RF has been proved to adequately meet the need for accuracy^18^. While separately quantifying the contribution of diverse influencing factors to the target value^4^, RF model can also intuitively present the independent and nonlinear correlation of the factors to thermal environment changes. In this study, with the spatial morphological parameters as the independent variable and the average LST of the unit as the dependent variable, the key factors affecting LST under each grid scale were analyzed and identified using the RF algorithm. Then, sensitivity analysis of the dependence between key factors and LST was further conducted based on the partial difference plots (PDP) of RF model.

## Results

### Spatial distribution of LST

**Figure 3:**
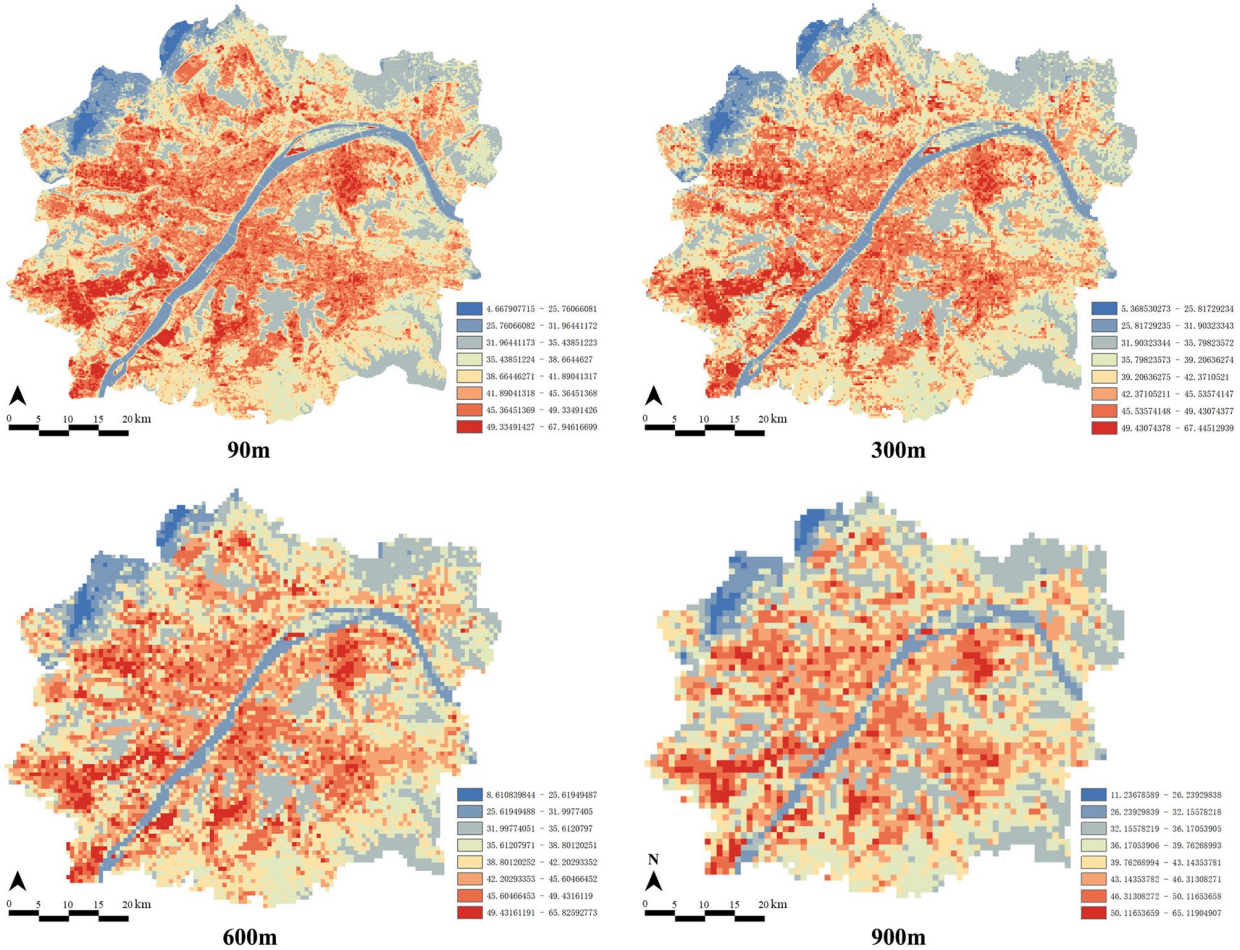
Spatial distribution of LST under different spatial grid size (Maps were generated using ArcGIS 10.5 (https://www.esri.com/en-us/home) and Adobe illustrator 2021 (https://www.adobe.com)).

According to the four different spatial grids of 90, 300, 600 and 900 m, the spatial distribution of LST over the study area was extracted respectively on ArcGIS 10.5 (Fig. 3). Consistent with the urban development pattern of Wuhan city, there were obvious differences of the LST spatial distribution within the WMA. Among them, the LST in the downtown area was higher than that in the suburbs. In the northeast and southwest of the city, several dispersed hot spots were deviated from the urban center. In addition, the areas with low LST in the city were mainly along the Yangtze River, followed by large greens space and urban lakes. Additionally, there occurred obvious differences in spatial heterogeneity of LST under different grid scales (Table 2). With the increase of spatial scale, the maximum and minimum LST decreased and increased respectively. At 900 m, the minimum LST was more than twice that of 90 m. Meanwhile, it can be found that the SD of LST also decreased along with the spatial scale, indicating that the spatial heterogeneity of LST was gradually weakened.

**Table 2:**
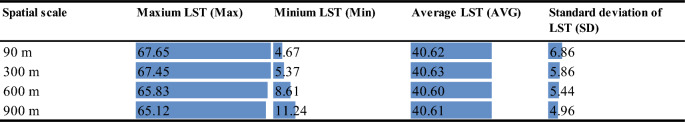
Max, Min, Avg and SD value of LST distribution at four spatial scale.

### Model performance

Since the spatial factors and LST were measured in square grids which may affect the representativeness of climate units, the performance for RF regression model needed to be determined before quantitatively analyzing the relative importance of multiple urban spatial morphological characteristics on thermal variations. It should be noted that in ML, the “training data” and “test data” are two subsets of data used for evaluating the model performance. The training set usually accounts for the majority of the total data set and is used to train the machine learning model. The test set is usually a small proportion of the total data set and is used to calculate the overall determination coefficient (R^2^) and root-mean-square error (RMSE) of the model to evaluate the accuracy of the model. Combined with previous studies^4,17^, the training set in this study accounted for 70% of the total data set and the test set accounted for 30% of the total data set. Accordingly, two valuation indicators including the R^2^ and RMSE of the dataset were conducted to quantify the accuracy of the model. The higher the R^2^, the lower the RMSE, and the more accurate the model. The experimental results for the four scales are shown in Table 3.

**Table 3:** R^2^ and RMSE of RF model's training dataset and test dataset among different spatial scales.

	R^2^				RMSE			
	90 m	300 m	600 m	900 m	90 m	300 m	600 m	900 m
Training data	0.685	0.796	0.891	0.593	1.587	1.294	1.061	1.355
Test data	0.362	0.625	0.684	0.440	2.180	1.813	1.468	1.679

According to the assessment results of R^2^ and RMSE, it can be found that the model had the high prediction accuracy at 300 and 600 m spatial scales. Especially at the 600 m neighborhood scale, the R^2^ of the model training dataset and the test dataset reached the highest value, which were 0.891 and 0.684, respectively. However, at a residential area (90 m) and community (900 m), model prediction accuracy was relatively low. In specific, the R^2^ of model training set and test set at 90 m scale were only 0.685 and 0.362.

### Relative influences of multiple building spatial factors to LST

**Figure 4:**
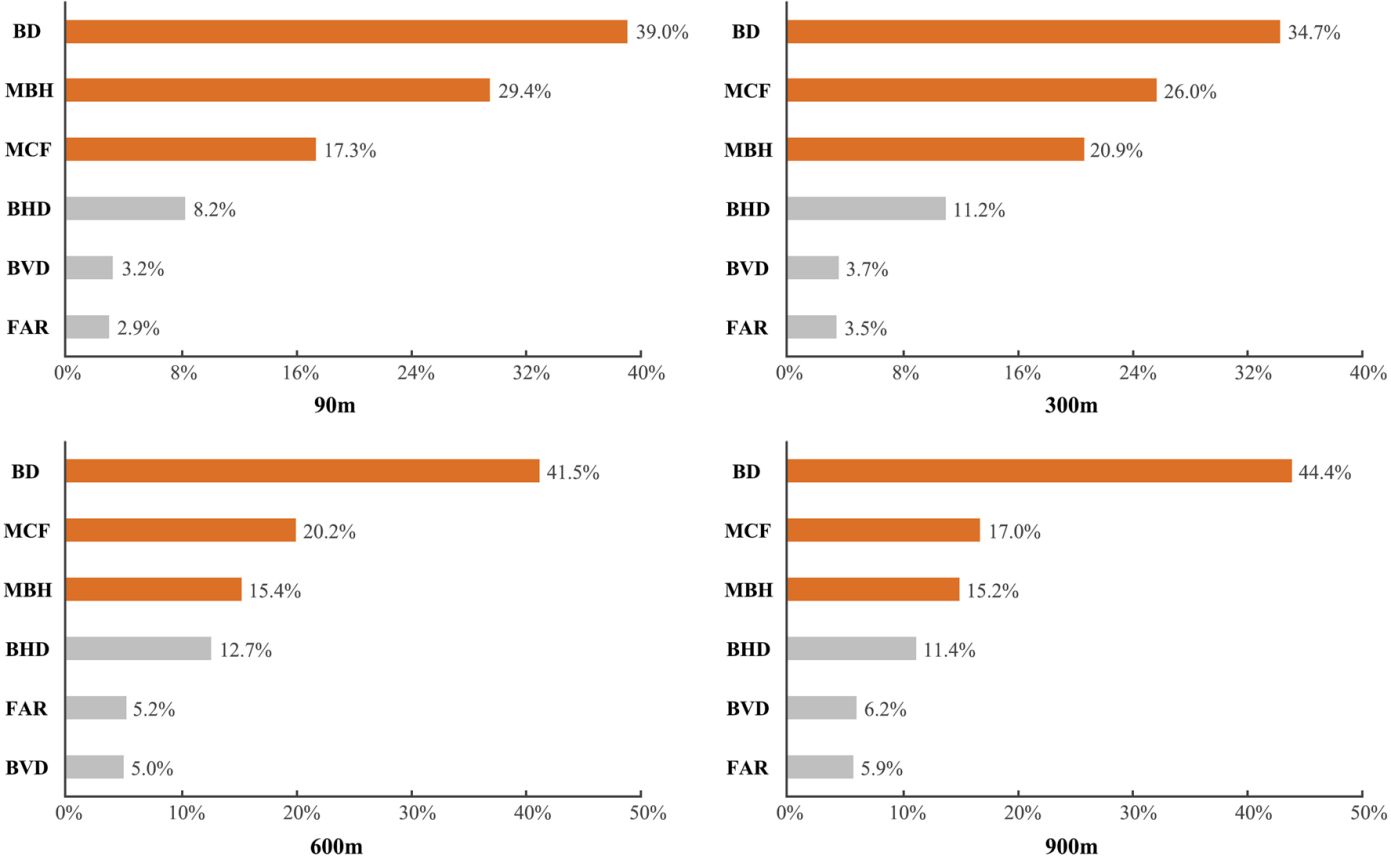
Sorting chart of the relative importance of the six factors in each scale. (**a**) 90 m grid scale, (**b**) 300 m grid scale, (**c**) 600 m grid scale, (**d**) 900 m grid scale.

At 90, 300, 600 and 900 m grid scales, the relative importance of six factors to LST was shown in Fig. 4. There were three factors that consistently had a relative importance over 15% for the LST variations in all grid scales, namely BD, MBH and MCF. Among them, BD ranked first and contributes more than 40% to the changes of LST. This suggested that BD should be the dominant spatial factor to drive the spatial heterogeneity of LST. MCF was the second most important variable at 300, 600 and 900 m grid scale, contributing nearly 20% to LST variation, but it was exceeded by MBH at 90 m grid scale. Compared with the above three factors, the BHD, FAR and BVD all had relatively little effect on the urban LST variation. In particular, the contributions of FAR and BVD were generally kept under the 5%, indicating that the influence of building height variation and 3D spatial volume on urban surface temperature was limited.

### Sensitivity analysis of key spatial factors on LST

**Figure 5:**
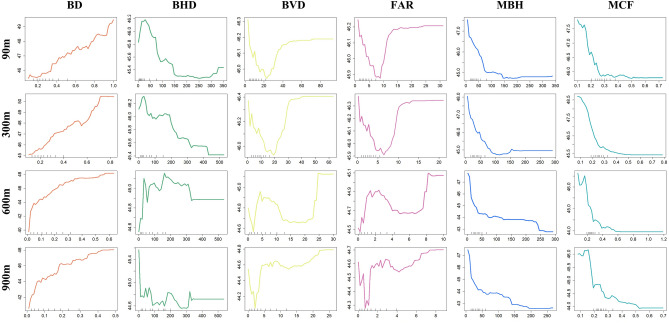
The partial dependence plots of LST on important input variables at different grid scales.

The influence of building forms on urban surface temperature was further analyzed, and the complex correlation models between them were revealed by using the PDP based on the RF results. The PDPs can reflect the variation of LST along with the spatial parameters, where the blue curve in Fig. 5 indicated the mean marginal effect. Intuitively, the PDPs of key factors including BD, MCF and MBH showed relatively stable trends in the four different grid scales, while the PDPs of other variables varied significantly in different scales.

It can be found from Fig. 5 that the LST was positively correlated with BD, and the correlation patten between them showed some variations within the different spatial scale. Specifically, BD and LST showed near-linear changes for 90 and 300 m. At 600 and 900 m, the change slope of LST gradually decreased with the increase of BD. When BD increased from 0 to 5%, LST risen rapidly, and then the slope gradually dropped and stayed flat after 40%. As the second significant variable, the effect of MCF on LST was stable and nonlinear. The results showed that the LST decreased rapidly when the MCF exceeded about 0.15. When MCF continued to exceed 0.5, LST decreased slowly or even remained unchanged with the increase of MCF. Similarly, the correlation pattern between MBH and LST was also nonlinear. With the increase of MBH, the LST started to drop sharply and then stabilized after 50 m. It was worth noting that at the 600 m and 900 m spatial scales, there was a drop in LST as MBH further increased to about 200 m. In addition, for BHD, BVD and FAR which had low contribution to LST changes, the correlation patterns between these variables and LST usually had multiple unstable turning points, making it ambiguous to predict the influence of corresponding spatial forms on LST. In general, correlation patterns of key factors and LST were summarized in Table 4. Among them, BD have a warming effect, while the other two factors show the nonlinear cooling effects on urban thermal environment.

**Table 4:** Correlation patterns in confidence intervals of the top 3 influencing factors and their maximum effect (°C) on LST.

Factor	BD (+)	MBH (−)	MCF (−)
Pattern			
	Linearly ascent	Gradual descent	Gradual descent
90 m grid scale	4.1591	− 2.8469	− 2.0594
300 m grid scale	5.2594	− 3.3492	− 3.2215
600 m grid scale	8.1792	− 5.0591	− 2.6948
900 m grid scale	7.6943	-4.9217	-2.3481

(+) means positive correlation, while (−) means negative correlation.

## Discussion

### The impacts of building morphology characteristics on LST

Using the RF regression algorithm, it was seen that relative thermal contribution of spatial forms on thermal environment highly differed, which was consistent with the conclusion of Wang et al.^4^. Accordingly, we first identified the key influencing factors that have significant effects on LST variation among the continuous spatial scales. Among them, the BD played the dominant role in driving the LST, which could explain more than 34.7% of its variance. Higher BD means more heat storage and fewer evaporation^4,28^, and the increase of its value can lead to a maximum increase of nearly 10 °C in LST. For the spatial grids of 300 m and above, the MBF which comprehensively characterize building vertical and horizontal size showed a stronger effect on LST change than the MBH. The main reason was that a higher MBF indicated a smaller volume of each building, which can enlarge the shadow coverage more efficiently. In addition, the FAR and SVD, which reflected the three-dimensional volume of building clusters, were the least correlated to LST changes.

The correlation between spatial forms and LST showed diversified patterns. According to the PDP results, it can be generally found that the 2D category of indicators represented by BD had a dominant and near-linear effect on LST warming, while MBH and MCF, which reflect the spatial vertical characteristics, have a certain effect on ground temperature dissipation. In specific, the six spatial factors can be categorized into two typical types: scale-varying factors (FAR, SVD and BHR) and scale-stable factors (BD, MBH and MCF). For scale-varying factors, their impacts on LST were highly complex, presenting many unstable transition points varying along the spatial scales. For the scale-stable factors, BD showed the linear and positive correlation to LST, which was consistent with the previous studies^18^. MBH and MCF both showed the consistent nonlinear correlation to LST, indicating that when the extent of the spatial index was larger than a threshold value, their cooling effect started to drop slowly. Specifically, the threshold value of MBH corresponded to 40–60 m, while the turning point of MCF was generally maintained at 0.3.

Furthermore, the spatial scales suitable for analyzing the thermal effects of spatial morphology at the grid size of 90, 300, 600 and 900 m were discussed in this study. It is found that the spatial forms were highly correlated with LST at 300 and 600 m scales, especially the 600 m. The reason could be that within the 600 m space range, the adjusted portion of its internal boundary layer lies entirely within the zone and does not overlap with surrounding^29^, which can make the thermal environment at this spatial unit relatively independent.

### Implication for urban heat mitigation

The Regulation and modification towards the urban surface structures have been proved an effective measure to deal with urban high temperature^23^. For building clusters, the existing strategies were mainly developed based on the linear relations between spatial parameters and thermal environment^30^, such as reducing the average height of buildings by 10 m to achieve a 1.9 °C reduction in the maximum heat island intensity^19^. However, more recent efforts have found that the various urban spatial forms were interdependent and can holistically affect the thermal environment, making it still ambiguous to determine the priority factors in realistic design scenarios^23^.

Here, it is theoretically suggested that the spatial unit with 600 m can be the suitable scale to predict the impacts of building morphology on LST changes with high confidence. From the perspective of urban planning and design, this size corresponds to an urban neighborhood which is bounded by street networks. Whitin a neighborhood, the buildings and network are more likely to form a unit with representative typology which significantly differed from the surrounding area, and it is conducive for planners to formulate the unit-based morphology management strategies^31^. Therefore, it can be reasonably inferred that the neighborhood units with the size of nearly 600 m can meet the accuracy of thermal environment research and design needs to a certain extent. It should be noted that the urban fabric is highly complex and characterized by continuous neighborhood units with the varying sizes and shape patterns. Thus, dividing the oversized neighborhood units as well as integrating fragmented small neighborhood units for flexibly approaching 600 m can be considered for better LST investigation and optimization purposes. On this basis, BD and MCF which reflect the spatial features of building coverage and compactness are the key control factors, followed by MBH. In most cases, restricting the BD and raising the building height, especially above 60 m, is an effective way to alleviate the local thermal environment^16,18^. Meanwhile, the arrangement of small-size buildings to further increase the shadow coverage should also be considered in actual practices.

### Further study

Although the urban building clusters are the primary cause to change the local environment and distinguish it from the natural conditions^32^, the thermal effects of other urban spatial elements such as surrounding greenery and water bodies also needs evaluation. However, natural spaces such as urban tree, grass, shrub and fragmented small water bodies do not have access to spatial information with the same level of high-precision as 3D building clusters, hindering the elaborate analysis of the impacts of diverse underlying surface materials and their spatial morphology on urban thermal environment. Therefore, subsequent research can focus on high-resolution remote sensing (such as airborne thermal imagery) to reveal the dependences between the urban characteristics and spatio-temporal heterogeneity of thermal environment. In addition, the design strategies related to ground heat dissipation developed in Wuhan can be inevitably limited because we mainly focus on the main environmental issue of urban heat mitigation during the hot summer. In order to delineate widely accepted adaptive design strategies, more evidences studies can be conducted in other regions with different spatial fabrics and climate background.

## Conclusion

In this study, the RF regression model was used to depict the comprehensive effects of complex building spatial forms on LST in Wuhan urban development area, and the key influencing factors with their influences were identified. Three conclusions can be made from the results. Firstly, we found that the 600 m neighborhood was the optimal analytical scale for investigating the thermal effects of building spatial forms and formulating the corresponding management and control strategies. Secondly, by quantifying the relative importance of multiple spatial forms to LST, BD, MCF, and MBH were highly correlated to LST and BD showed the greatest impacts. Third, the partial dependence plots presented the LST change along with the key influencing factors, and their correlation patterns differed. Among them, BD showed a positive and linear correlation with LST. On the contrary, the MCF and MBH showed the negative and non-linear pattern, in which the obvious turning point appeared. These findings suggested that controlling building density and keeping the areas relatively open was the primary issue to mitigate the high temperature. Under the same housing demand, raising the building height appropriately and restrict large-size buildings should also be considered to further reduce the urban surface temperature. Furthermore, this study will provide a reference for a better understanding of the spatial heterogeneity of LST and the establishment of climate adaptive countermeasures.

## Data Availability

The datasets used and/or analysed during the current study available from the corresponding author on reasonable request.
